# Use of color Doppler ultrasound to differentiate Graves’ disease from silent thyroiditis in patients treated at Fundación Valle del Lili in 2022-2024

**DOI:** 10.3389/fendo.2026.1748502

**Published:** 2026-04-16

**Authors:** María Bernarda Iriarte Durán, Oriana Arias Valderrama, Francisco Javier Meza Díaz, Mayra Alejandra Rojas Ramirez, María Angélica Guerra Soto, Andrés Octavio García-Trujillo, Flor María Medina, Luz Ángela Casas-Figueroa

**Affiliations:** 1 Facultad de Salud, Departamento de Medicina, Universidad Icesi, Cali, Colombia; 2 Centro de Investigaciones Clínicas, Fundación Valle del Lili, Cali, Colombia; 3 Fundación Valle del Lili, Departamento de Medicina Interna, Servicio de Endocrinología, Cali, Colombia; 4 Fundación Valle del Lili, Servicio de Radiología e Imágenes Diagnósticas Carrea, Cali, Colombia

**Keywords:** antibodies against TSH receptor, Graves’ disease, silent thyroiditis, thyroid doppler, thyroid scintigraphy

## Abstract

**Background:**

Graves’ disease (GD) and silent thyroiditis (ST) are common etiologies of thyrotoxicosis requiring optimal diagnosis to provide different therapeutic approach. Here we compare the diagnostic performance of color Doppler thyroid ultrasound (CDU) with measurement of thyroid artery systolic velocities, thyroid scintigraphy with Tc99 and anti-thyroid-stimulating hormone receptor antibodies (TRABs) to differentiate GD from ST in a tertiary center in Cali, Colombia, between 2022-2024.

**Methods:**

This single-center prospective study included 78 adult patients diagnosed with thyrotoxicosis between January 2022 and October 2024. Participants had available information on medical records, thyroid function tests, thyroid peroxidase antibodies, TRABs, CDU and, thyroid scintigraphy at the time of diagnosis. Data analysis was performed in October 2024 and determined area under the curve, sensitivity, and specificity of CDU and scintigraphy for differentiating GD from ST, and the performance of each measured parameter were determined.

**Results:**

78 patients were included, 63% were diagnosed with Graves’ disease, and 37% with thyroiditis. 28/29 patients with thyroiditis (96.6%) had superior thyroid artery systolic velocities (STV) < 43 cm/s and 25/29 of cases (86.2%) had thyroid tissue blood flow (TBF) < 14.1%. The presence of a diffusely increased pattern + percentage of trapping higher than 4.9% in scintigraphy had a sensitivity of 80% and a specificity of 90% for the diagnosis of GD.

**Conclusions:**

CDU of the thyroid arteries with measurement of systolic velocities is a useful diagnostic tool for differentiating between GD and ST. A cut-off point of STV < 43 cm/s has a high positive predictive value for ST diagnosis.

## Introduction

Thyrotoxicosis is prevalent in the general population (1.3%), with Graves’ disease (GD) and silent thyroiditis (ST) being the most common etiologies (1, 2), in addition to being clinically similar (3–5). The differential diagnosis of these two entities is made by measuring antibodies against the TSH receptor (TRABs) and obtaining characteristic thyroid scintigraphic patterns (2, 4). However, these methods are not always available in lower-middle income countries, and results may take a long time to obtain. To address these limitations, color doppler ultrasound (CDU) of the thyroid can be a fast, inexpensive, and noninvasive diagnostic method that facilitates therapeutic decisions in these patients (6).

Increased flow in the superior and inferior thyroid arteries has acceptable sensitivity and high specificity for the diagnosis of Graves’ disease (7–9). This is not a new concept, however there are few studies which assess the performance of CDU in this setting. A meta-analysis of 11 Asian studies, with 1052 patients, published in 2019 by Peng et al. (6), found that an increased peak systolic flow in the superior thyroid artery has a sensitivity of 86% (95% CI, 0.80-0.90) and specificity of 93% (95% CI, 0.86-0.97) to distinguish GD from ST, with an area under the curve (AUC) of 0.94 (95% CI, 0.92-0.96), a positive likelihood ratio of 13.0 (95% CI, 6.1-27.8) and a negative likelihood ratio of 0.15 (95% CI, 0.10-0.22). This meta-analysis showed moderate heterogeneity (I^2^ = 65.7%) with variable peak systolic velocity cut-off points between 40 and 50 cm/s (6), the population under evaluation were in Asian countries and number of participants (1052) did not allow subgroup analysis.

In Latin America, a study carried out in Brazil with 176 patients with thyrotoxicosis showed that CDU had a sensitivity of 96% and 72.7% for the diagnosis of diffuse toxic goiter (generally secondary to GD) in symptomatic and asymptomatic patients, respectively (10); on the other hand, the sensitivity for the diagnosis of ST in this same study was 95% (10). Despite the evidence presented, the evaluation of thyroid vascularization is rarely used in low-income countries, largely due to the absence of local studies that prove its diagnostic performance and because guidelines don´t agree the cutoff point in STV and ITV with best performance for differentiating GD *vs*. ST (11).

Evidence on CDU performance is growing, but management guidelines do not clarify the ideal STV or ITV cutoff point for GD diagnosis. Another meta-analysis to elucidate diagnostic thresholds of peak systolic flow velocities in thyroid arteries for the discrimination of GD and ST was published in 2024 (12). A total of 18 studies (prospectives and retrospectives) with 1276 participants were analyzed and results support a STV above 68.63 cm/s for diagnosis of GD with up to 91% efficacy. It should be noted that some of these studies included healthy patients, and to facilitate comparisons with other groups, authors combined the data from STV and ITV in normal participants, resulting in a combined cutoff value. When analyzing the data, it is important to emphasize that the heterogeneity of the studies was greater than 80%.

Additionally, previous publications do not necessarily address the everyday clinical scenario we face, in which we have patients with an intermediate probability of one or the other disease and in whom an early diagnostic test really makes a difference in the treatment and prognosis of the disease. For example, in a survey of endocrinologists in Colombia (13), only 20% of the participants requested TRABs in the first evaluation of patients with hyperthyroidism, probably due to poor access to the measurement of these antibodies in several regions from a local point of view, but also in other countries around the world. This finding supports the marked differences in diagnostic and therapeutic interventions in patients with hyperthyroidism (14, 15).

Further, thyroid ultrasound plays a key role in evaluating the size and vascularization of the gland in patients with hyperthyroidism, allowing to differentiate between toxic adenoma and GD, whose therapeutic approach and recurrence rates are different (5, 16, 17). Also, the characterization of the number, size, and location of thyroid nodules is another advantage because the prevalence of thyroid nodules in the population varies between 19-67% (2, 18) and their detection influence decision-making and appropriate follow-up management plan (19).

Based on these considerations, the main objective of our study was to determine the diagnostic performance of thyroid CDU with measurement of thyroid artery systolic velocities compared to Tc99 scintigraphy and TRABs in differentiating GD from ST.

## Materials and methods

### Study population

This was a study of diagnostic accuracy with prospective data collection. We consecutively included 78 patients (age range 34–48 years) presenting to the emergency service, outpatient clinics, and hospitalization units with a diagnosis of thyrotoxicosis prior to initiation of any treatment. This design was chosen to ensure systematic and standardized data collection and to allow evaluation of diagnostic accuracy under real-world clinical conditions. This study was approved by the Institutional Review Board (IRB) of Hospital Universitario Fundación Valle del Lili (Cali, Colombia) under protocol registry number 1854-2022. Informed consent was obtained from all participants prior to enrollment, in accordance with the ethical principles outlined in the Declaration of Helsinki. Data analysis was performed in October 2024 and included participants with complete available information on thyroid function tests, thyroid peroxidase antibodies (TPO), TRABs, CDU of the thyroid, and thyroid scintigraphy at the time of diagnosis.

The inclusion criteria were: (1) Adults > 18 years with diagnosis of HPT confirmed through serologic testing, including FT3, FT4, TSH, and TRAb; (2) availability of STV or ITV measurements in CDU; (3) scintigraphic evaluation in all patients and (4) clear categorization of study participants into groups (GD and ST). Exclusion criteria were patients under 18 years of age, pregnant women, patients with hemodynamic instability due to the risk of developing thyroid storm when discontinuing antithyroid drugs and beta blockers; patients who refused to participate or unwillingness to undergo CDU or scintigraphy; prior intake of iodinated contrast media within the preceding 3 months; current or recent use of amiodarone; and the presence of contraindications to any of the study procedures. We exclude other causes of thyrotoxicosis such as toxic adenoma.

### Clinical evaluation

The diagnosis of HPT followed the recommendations of current clinical practice guidelines (3,5). Patients taking antithyroid drugs were subjected to a washout period of seven days before scintigraphy evaluation (16, 17), and the wash out period was 48 hours for patients taking beta blockers (20). Participants were closely monitored during the washout period for these medications, and no adverse effects were observed when they were temporarily discontinued. Information about sex, race, age, medical history, medications, and symptoms was recorded in the electronic medical charts. The presence of orbitopathy or dermopathy, blood pressure (BP), and heart rate was obtained through physical evaluation at the time of diagnosis.

### Biochemical evaluation

All participants underwent measurements of the following parameters by chemiluminescence immunoassay ([CLIA], Alinity ci-series core laboratory, Abbott): pregnancy test (if women of childbearing age), thyroid stimulating hormone (TSH), free thyroxine (fT4) levels, total triiodothyronine (tT3), TPO antibodies, and TRABs. HPT was defined by TSH levels below 0.1 µU/mL, with fT4 levels above 1.7 ng/dL and/or tT3 above 2.0 ng/mL. Positive TPO antibodies were considered at levels higher than 5.61 UI/mL and the gold standard test for the diagnosis of Graves’ disease was TRABs levels higher than 1.75 UI/mL.

### Radiological and scintigraphic evaluation

CDU was performed on patients at supine position after 10 minutes of rest with Aplio i700 (TUS-AI700) (Canon Medical Systems, Otawara, Tochigi, Japan) with iDMS (intelligent Dynamic Micro-Slice) Matrix technology Linear probe PLI-1205BX (ultra-wideband 4.0 to 18.3 MHz) and linear probe PLT-1005BT (widband 3.8 to 14.00 MHz) (9). The superior and inferior thyroid arteries were assessed in the transverse plane, that is, crossing the common carotid arteries, and in the longitudinal plane, that is, parallel to the common carotid arteries (21). The peak flow velocity (PSV) in each artery was measured and averaged to obtain a single value for the superior thyroid arteries (STV) and another for the inferior thyroid arteries (ITV). A cut-off point of 43 cm/s was taken to distinguish between Graves’ disease and silent thyroiditis (3, 6, 22).

Additionally, the ADF function (Advanced Dynamic Flow) was used after activating the TDI key for measuring thyroid tissue blood flow (TBF) (23), expressed as a percentage. The last parameter is the average systolic flow velocity in the common carotid arteries (CCV) to confirm the correct positioning of the probe (9, 21). The assessment was conducted by one experienced radiologist (F.M) who had more than 5 years of experience and was blinded to the etiology of thyrotoxicosis.

Thyroid scintigraphy with 99mTc-pertechnetate (Tc99) was performed 20 min after the administration of 5 mCi of Tc99 (17, 24). Images of the anterior neck region were obtained for 5 min using a gamma camera with a low-energy collimator. Entrapment percentages between 2.5 and 4.5% were considered normal (24).

### Statistical analysis

Study data were collected and managed using REDCap (Research Electronic Data Capture), a secure, web-based software platform designed to support data capture for research studies, hosted at Hospital Universitario Fundación Valle del Lili. REDCap provides an intuitive interface for validated data entry, audit trails for tracking data manipulation and export procedures, and automated export functionality compatible with common statistical software packages. Patient anonymization was ensured throughout the data collection process, and data quality was monitored by means of built-in validation rules and double-entry verification procedures.

Sample size was estimated for the comparison of proportions, based on the diagnostic performance of the reference standard which has a reported sensitivity of 97%. Assuming a minimum detectable sensitivity of 80% for color CDU, with an acceptable margin of error of 10% and a 95% confidence level, a minimum sample size of 110 participants was estimated to be required. Enrollment was stopped at 78 patients due to logistical and time constraints within the study period (January 2022 – October 2024). A *post-hoc* sensitivity analysis confirmed that the observed difference in proportions between CDU and scintigraphy reached statistical significance, acknowledging that this represents a limitation of the study in terms of statistical power.

Categorical variables were compared between groups using Pearson’s Chi-squared test, or Fisher’s exact test when expected cell frequencies were less than 5. For continuous variables, between-group comparisons were performed using the Wilcoxon rank-sum test (Mann-Whitney U test), given that normality assumptions were not met or could not be assumed. A two-tailed p-value of less than 0.05 was considered statistically significant.

Univariate analysis was used to verify the distribution of the data of the numerical variables, using the Shapiro-Wilk test, with a p-value less than 0.05, which was considered significant. Numerical variables with normal distribution were summarized using means and standard deviations; variables without normal distribution were expressed as medians and interquartile ranges. Qualitative variables are summarized as proportions and presented in frequency tables. The area under the curve, sensitivity, and specificity of CDU for differentiating between Graves’ disease and silent thyroiditis, and the performance of each measured parameter were determined using R Studio software version 4.3.3.

## Results

### Baseline characteristics of the cohort

A total of 49 participants were diagnosed with Graves’ disease (63%), and 29 with thyroiditis (37%). Proportion of female patients were 60/78 (77%) and the most frequent comorbidities in the cohort were arterial hypertension (14%), anxiety disorders (14%), type 2 diabetes mellitus (7.7%), and obesity (6.4%). 57/78 patients (73%) manifested symptoms of thyrotoxicosis on admission. The biochemical profiles of participants are shown in **Table 1**.

**Table 1:** Cohort characteristics.

Variable	N = 78^1^
Age (years)	41 (34-48)
Race	
Mestizo	36 (46%)
Mulato	1 (1.3%)
Afro-American	12 (15%)
Caucasic	29 (37%)
Gender	
Female	60 (77%)
Male	18 (23%)
Thyrotoxicosis Symptoms	57 (73%)
Beta-blockers Use	20 (26%)
Thyroid orbitopathy	9 (12%)
Thyroid dermatopathy	0 (0%)
Graves’ disease	49 (63%)
Thyroiditis	29 (37%)
TSH (µUI/ml)	0.01 (0.01-0.01)
T3L (ng/mL)	1.82 (1.27-2.68)
T4L (ng/dL)	2.20 (1.73-3.24)
TPO antibodies (UI/mL)	113 (3-648)
TRABs (UI/mL)	4 (1-14)

^1^median (IQR); N (%).

### Performance of thyroid CDU parameters for diagnosis of Graves’ disease *vs*. silent thyroiditis

**Table 2** shows the values obtained for the different ultrasound parameters in patients with Graves’ and thyroiditis. Statistically significant differences were found in the values of STV, ITV, and TBF, which were higher in patients with Graves’ disease (p < 0.001). 28/29 patients with thyroiditis (96.6%) had an STV < 43 cm/s and 25/29 of these patients had TBF < 14.1% (86.2%).

**Table 2:** Performance of color Doppler thyroid parameters for diagnosis of GD *vs*. ST (based on reference standard, TRABs).

Parameter	Graves, N = 49^1^	Thyroiditis, N = 29^1^	p-value^2^
STV			
≥ 43 cm/s	29 (59%)	1 (3.4%)	<0.001
< 43cm/s	20 (41%)	28 (96,6%)	<0.001
ITV (cm/s)			
≥ 43 cm/s	25 (51%)	1 (3.4%)	<0.001
< 43cm/s	24 (49%)	28 (96,6%)	<0.001
CCV (cm/s)	85 (73-104)	75 (64-100)	0,072
FBT	16% (12-26)	8% (5-12)	<0.001

Cut-off point 14.1 for FBT	Graves, N = 49^1^	Thyroiditis, N = 29^1^	p-value^2^
> 14.1%	30 (61%)	3 (10,4%)	<0.001
≤ 14.1%	16 (33%)	25 (86,2%)	<0.001
Missing data	3 (6%)	1 (3,4%)	–

^1^n (%); median (IQR).

^2^Pearson’s Chi-squared test; Wilcoxon rank sum test STV - Superior Thyroid Artery Systolic Velocities, ITV - Inferior Thyroid Artery Systolic Velocities, CCV - Mean Systolic Velocities of Common Carotid Arteries, FBT - Thyroid Tissue Blood Flow.

**Figure 1:**
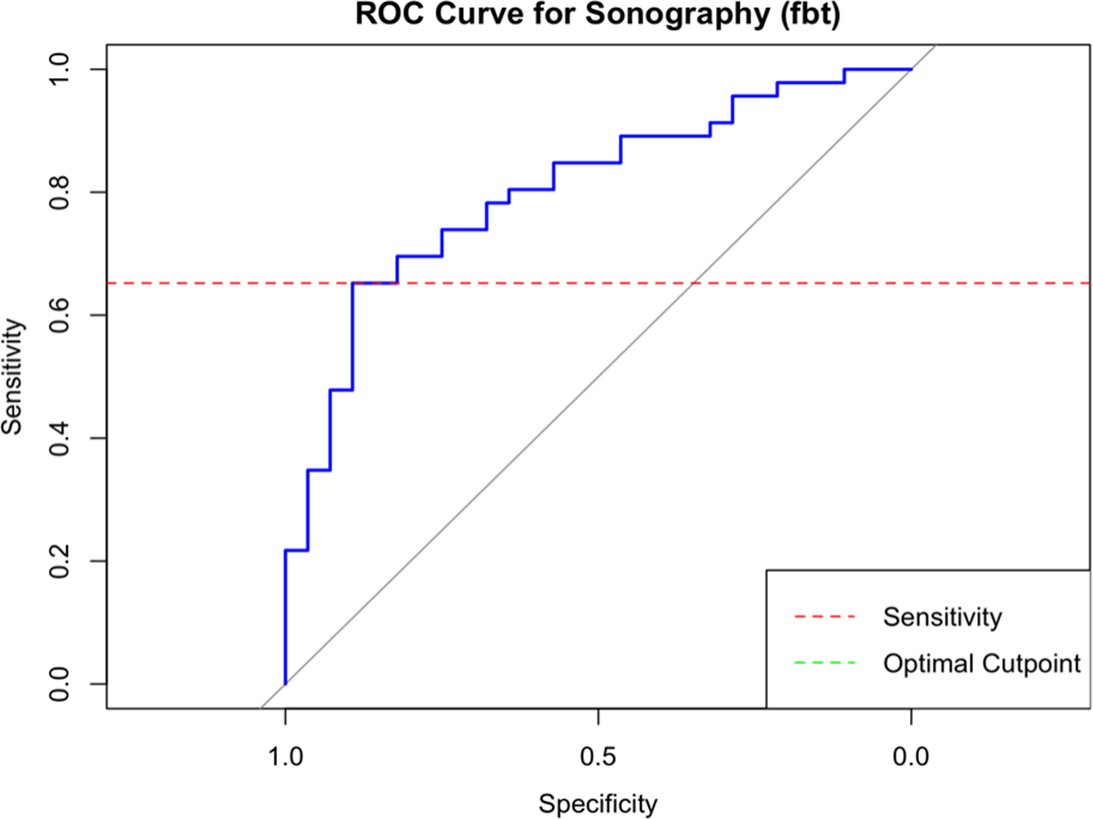
ROC curve for thyroid tissue blood flow (TBF) in CDU Threshold 14.15%: Specificity 89%, sensitivity 65%, area under the curve (AUC): 0.80 for diagnosis of GD.

The threshold for TBF was determined to be 14.1% with a sensitivity of 65% and specificity of 89%. Based on the reference standard (TRABs), a sum STV > 43 cm/s and TBF > 14.1% had a sensitivity of 41% and specificity of 97% for the diagnosis of Graves’ disease, with a PPV of 95% and NPV of 49% (**Table 2**). **Figure 1** shows the ROC curve for thyroid tissue blood flow in CDU (TBF).

### Performance of scintigraphic parameters for diagnosis of Graves’ disease *vs* silent thyroiditis

**Figure 2:**
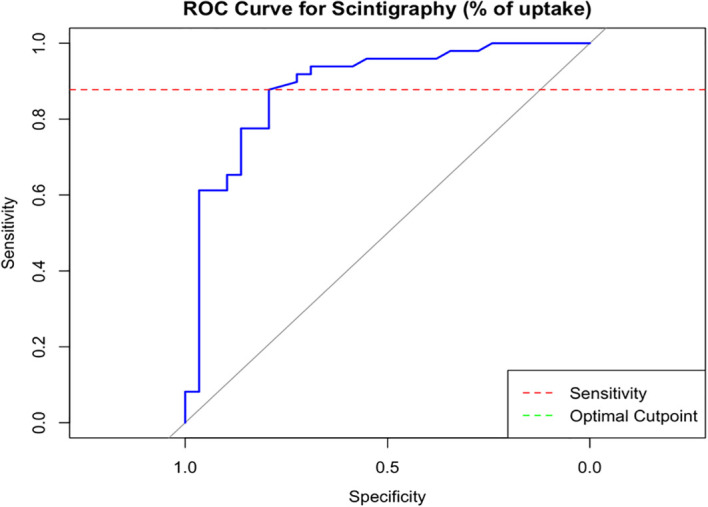
ROC curve for percentage uptake in scintigraphy. Threshold 4.9%: Specificity 79%, sensitivity 88%, area under de curve (AUC): 0.88 for diagnosis of GD.

A diffusely increased pattern was present in 42/49 of patients with Graves’ disease (86%), with the percentage of trapping being higher in these participants than in those with thyroiditis (median 14% *vs*. 2%, p < 0.001). The presence of a diffusely increased pattern + percentage of trapping > 4.9% had a sensitivity of 80% and specificity of 90% for the diagnosis of Graves’ disease, with a PPV of 93% and NPV of 74%. **Figure 2** shows the ROC curve for the percentage uptake on thyroid scintigraphy.

The statistical power of the estimates for ultrasound based on specificity was 99.5%, with a Positive Likelihood Ratio (LR+) of 11.84 and a Negative Likelihood Ratio (LR-) of 0.61, these likelihood ratios indicate a strong diagnostic performance (**Table 3**). On the other hand, the statistical power of the estimates for scintigraphy was 97%, with LR+ of 7.89 and LR- of 0.20, ruling out GD when the result was negative (**Table 3**).

**Table 3:** Performance of diagnostic tests in this study.

Test	US doppler	Scintigraphy	US doppler + scintigraphy
Sensitivity, %	41	82	41
Specificity, %	97	90	100
%PPV	95	93	100
% NPV	49	74	50
Positive LR	11.8	7.89	inf
Negative LR	0.61	0.20	59

PPV, positive predictive value; NPV, negative predictive value; LR, likelihood ratio.

## Discussion

Untreated hyperthyroidism leads to worse health outcomes, such as increased cardiovascular events, especially a higher incidence of atrial fibrillation and embolic stroke, higher incidence of fractures, lower quality of life, and increased mortality compared to the general population (25–27). Clinical differentiation between mild GD and silent thyroiditis can be a diagnostic challenge due to the absence of classic signs, such as orbitopathy (which may be present in 20-30% of cases of GD) and skin changes (2, 4, 5). Therefore, guidelines for the management of patients with thyrotoxicosis recommend various diagnostic tests to clarify the etiology and institute timely management (3–5).

An international survey of clinical practice patterns in the management of GD published in 2024 by Villagelin et al. (28) showed that 77.4% of participants obtain TRAb measurement for diagnosis and 61.3% had US as an anatomical or functional image. Only 15.9% of cases had functional imaging like scintigraphy, which shows a change in clinical practice over the last decade. It was a self-reported survey, so there may be a selection and reporting bias, although we do not know if some countries are overrepresented. Certainly, the transition in clinical practice standards globally is influenced by resource availability and evidence-based medicine, knowing that latter factor has not demonstrated complete validation of a precise cutoff point for thyroid artery systolic velocities.

The use of antithyroid drugs for the management of thyrotoxicosis secondary to GD is the first-line treatment in most cases, which achieves rapid control of hyperthyroidism with an acceptable safety profile (29, 30). Antithyroid drugs are known to be ineffective in patients with thyroiditis and may favor the development of severe hypothyroidism and even myxedema crisis (31). Therefore, clarifying the etiology of thyrotoxicosis before treatment initiation is crucial.

Currently, the wide availability of immunoassays with high sensitivity for the measurement of TRABs makes this laboratory test key to confirming GD (32). However, immunoassay does not identify the functionality of the antibodies (stimulating or blocking) on the TSH receptor, which is important in predicting remission or relapse after completing a course of antithyroid medication (33, 34). Other disadvantages are the cost of the test, time consumption to obtain the results and negative result does not confirm silent thyroiditis especially in the presence of undiagnosed nodular thyroid disease (2, 4, 32).

Historically, thyroid ultrasound has been used to detect thyroid nodules on physical examination or scintigraphy in patients with hyperthyroidism (2, 4). However, because thyroid blood flow is increased in patients with GD and decreased in those with various forms of thyroiditis, color Doppler is a tool with dual utility (structural and functional) (9). Measurement of the PSV of thyroid arteries, particularly the average of the superior arteries, provides quantitative information to distinguish between GD and thyroiditis (6). It is well known that access to thyroid arteries can be technically difficult, especially the inferior artery if one lacks experience. With good anatomical orientation and the ability to optimize the angle of insonation, ideally less than 60°, color and spectral Doppler with modern equipment can be achieved. This additional protocol is considered to require a moderate learning curve and is within the scope of conventional radiological training (35, 36).

Different cutoff points have been used for diagnosis in studies published to date that have sought to evaluate the performance of CDU. A retrospective study with 135 participants published in 2012 by Zhao et al. (37) showed that STV > 50 cm/s could distinguish GD from thyroiditis with a sensitivity of 81% and specificity of 96%, in addition to STV having an area under ROC like TRABs measurements. In our study we took a cut-off point for STV < 43 cm/s to rule out GD (specificity 96.6%) in alignment with previous studies as published by Hiraiwa et al. (7). This study observed that in cases of silent thyroiditis, STV rarely exceeds 40 cm/s, even in acute phases, and that cases of GD usually present STV values greater than 43 cm/s. To determine the best NPV and avoid false positives, our study evaluated additional ultrasound parameters, among these TBF that provides specificity of 89%, sensitivity of 65% and AUC: 0.80 for diagnosis of GD.

A cross-sectional study on 111 patients with thyrotoxicosis published by Sarangi et al. in India (38), found that a mean STV greater than 54.3 cm/s had 82.9% sensitivity and 86.2% specificity for diagnosis GD. Interestingly, they calculated STV/CCA ratio to differentiate between GD and ST, a parameter less investigated in this field. The cutoff point of this parameter with best performance to support diagnosis of GD was > 0.4 (80.5% sensitivity and 86.2% specificity). This study supports the theory that different cut-off points in CDU parameters may be related to ethnicity. Regarding the biochemical evaluation of the participants, the authors confirmed that the performance of the FT3/FT4 ratio did not provide adequate discriminatory ability to differentiate GD and ST (38).

Recently, Morkos et al. from the Indiana University School of Medicine published a retrospective study (22) that evaluated the utility of CDU in patients with thyrotoxicosis. In this study, a PSV ≥ 40 cm/s in at least one thyroid artery was considered a reasonable cut-off for Graves’ disease. CDU was accurate in 89% of patients and provided the diagnosis during the initial encounter in 75% of patients before additional testing, along with screening for thyroid nodules. The median PSV in patients with GD was 44.1 cm/s, very similar to the cut-off point of our study for diagnosing GD. Morkos et al. took the highest PSV measured in the right and left thyroid artery, which can generate high variability in the data since the Doppler flow is not the same in these locations. Additionally, since this was a retrospective study, two different ultrasound machines were used, which may introduce biases in the results.

Another advantage of CDU is the identification of thyroid nodules, which allows the characterization and assessment of malignancy risk and can modify therapeutics in patients with thyrotoxicosis. In 2019, a meta-analysis published by Papanastasiou et al. analyzed 7 studies with 2582 participants (39). Patients with GD having at least one thyroid nodule were more likely to have thyroid cancer among those surgically treated (odds ratio [OR] 5.3, 95% confidence interval [CI] 2.4-11.6, I2 83%).

Although the coexistence of thyroid cancer and hyperthyroidism is rare (2-8%), a study published in 2009 with 325 participants determined the frequency of thyroid carcinoma (TC) in patients with thyrotoxicosis either due to GD, toxic adenoma (TA) or toxic multinodular goiter (TMNG) (40). Preoperative evaluation was performed by ultrasonography-guided fine needle aspiration biopsy. Participants with TMNG had more nodules compared to other etiologies of HPT, 87% of cytologies were benign, 6.2% inadequate, 4.3% suspicious and 1.9% malignant. The TC rate in patients with TMNG was 16%, 6.4% in TA and 12.6% in GD (40).

Our findings can motivate future sub-analysis of our study to determine the prevalence of thyroid nodules and the modification of treatment in these patients, based on the results of thyroid ultrasound. Likewise, it would be interesting to carry out cost-effectiveness studies with these three diagnostic tests (US + CDU, scintigraphy and TRAB measurement) not only for an accurate diagnosis of the etiology of thyrotoxicosis, but also to evaluate the cost-utility of different therapeutic strategies in patients with GD (surgical *vs*. long-term antithyroid management). It would be interesting to determine whether any of the CDU parameters could predict worse prognosis in patients with GD during follow-up and thus be able to offer early definitive management in these patients.

Finally, there are certain limitations to our study. It does not allow causality to be established due to its temporal nature. With the aim of determining the performance of CDU as a diagnostic test in a single center it is not possible to generalize the results to other populations. The sample size did not allow us to evaluate the statistical power of ultrasound estimates based on sensitivity. Because some patients with thyroiditis may have increased thyroid blood flow, especially during the hypervascular phase, we observed that 3.4% of cases had an STV > 43 cm/s and 11% had a TBF > 14.1.

## Conclusion

CDU of thyroid arteries is a useful diagnostic tool for differentiating between Graves’ disease and silent thyroiditis, both of which are common causes of thyrotoxicosis in our environment. Due to its excellent specificity, easy accessibility, safety, and low cost compared to thyroid scintigraphy and TRABs measurements has the advantage for be used in different scenarios during clinical practice (emergency room, general ward and outpatient setting). In our population, we found that a STV > 43 cm/s is an optimal cutoff point for differential diagnosis between GD and ST, a sum STV > 43 cm/s and TBF > 14.1% had a PPV of 95% for the diagnosis of Graves’ disease.

## Data Availability

The raw data supporting the conclusions of this article will be made available by the authors, without undue reservation.
